# Synthetic Morphology Using Alternative Inputs

**DOI:** 10.1371/journal.pone.0006946

**Published:** 2009-09-10

**Authors:** Hiromasa Tanaka, Tau-Mu Yi

**Affiliations:** 1 Department of Developmental and Cell Biology, University of California Irvine, Irvine, California, United States of America; 2 Center for Complex Biological Systems, University of California Irvine, Irvine, California, United States of America; University of Edinburgh, United Kingdom

## Abstract

Designing the shape and size of a cell is an interesting challenge for synthetic biology. Prolonged exposure to the mating pheromone α-factor induces an unusual morphology in yeast cells: multiple mating projections. The goal of this work was to reproduce the multiple projections phenotype in the absence of α-factor using a gain-of-function approach termed “Alternative Inputs (AIs)”. An alternative input is defined as any genetic manipulation that can activate the signaling pathway instead of the natural input. Interestingly, none of the alternative inputs were sufficient to produce multiple projections although some produced a single projection. Then, we extended our search by creating all combinations of alternative inputs and deletions that were summarized in an AIs-Deletions matrix. We found a genetic manipulation (AI-Ste5p *ste2Δ*) that enhanced the formation of multiple projections. Following up this lead, we demonstrated that AI-Ste4p and AI-Ste5p were sufficient to produce multiple projections when combined. Further, we showed that overexpression of a membrane-targeted form of Ste5p alone could also induce multiple projections. Thus, we successfully re-engineered the multiple projections mating morphology using alternative inputs without α-factor.

## Introduction

Cells respond to various extracellular chemical and physical inputs such as light, osmotic pressure, growth factors and neurotransmitters. Receptors detect the extracellular inputs, and then activate signal transduction networks that mediate specific output responses such as the transcription of genes (short-term response) or cellular morphological changes (long-term response). A synthetic approach is a powerful method to further the understanding of biological systems [1], and reproducing natural outputs without using the natural inputs is an important goal in synthetic biology.

The mating signaling network in budding yeast is one of the most well-analyzed signal transduction systems [2]. Haploid **a**-cells respond to the extracellular input α-factor to mate with α-cells. Transcriptional activation of mating-related genes, formation of mating projections, and fusion of two opposite mating type cells are involved in this process. Binding of the input α-factor to α-factor receptor (Ste2p) leads to activation of the heterotrimeric G-protein: Gα (Gpa1p) releases GDP, binds GTP, and dissociates from Gβγ (Ste4p/Ste18p). Free Gβγ recruits to the plasma membrane the scaffold protein Ste5p [3], [4], which tethers together the mitogen activated protein kinase (MAPK) cascade (Ste11p → Ste7p → Fus3p/Kss1p) for its signaling specificity [5]. Activated Fus3p phosphorylates the transcription factor Ste12p and its inhibitors Dig1p/Dig2p, resulting in the transcription of mating-related genes.

There are dramatic changes in cell morphology during the mating response. In particular, cells form a mating projection that arises from the combined actions of heterotrimeric G-protein, MAPK, and Cdc42 signaling, which regulate the spatial dynamics of the cytoskeleton, cell membrane, and cell wall [6]. Intriguingly, when cells are exposed continuously to high concentrations of α-factor, they will form multiple mating projections [7]–[9]. The mechanisms underlying this process are not fully understood, and characterizing this oscillatory behavior is an interesting challenge for systems and synthetic biology. It has been shown that certain loss-of-function mutations prevent this multiple projection phenotype, although the mutants can still make a single projection [9].

Here, we describe a novel approach to re-engineer the yeast mating morphology which we term “Alternative Inputs to α-Factor”. An alternative input (AI) is defined as any genetic manipulation that can activate the signaling pathway instead of the natural input. We addressed the question of whether alternative inputs could induce multiple projections or not. No single alternative input could induce multiple mating projections, although some produced a single projection. To broaden the search as well as to characterize the existing AI morphologies, we created all possible combinations of alternative inputs and deletions summarized in an AIs-Deletions matrix. Interestingly, we found that AI-Ste5p (overexpressed Ste5p) induced a polarized cell phenotype even in the absence of MAPK activity and transcriptional activation. In addition, we discovered a genetic manipulation (AI-Ste5p *ste2Δ*) that enhanced the formation of multiple projections. Pursuing this lead, we demonstrated that Ste4p and Ste5p were sufficient to produce multiple projections when overexpressed together. Finally, we found that overexpression of a membrane-targeted form of Ste5p alone could also produce multiple projections. Thus, we re-engineered the mating morphology using alternative inputs to induce multiple mating-projections without α-factor.

## Results

### Alternative Inputs to α-factor

**Figure 1:**
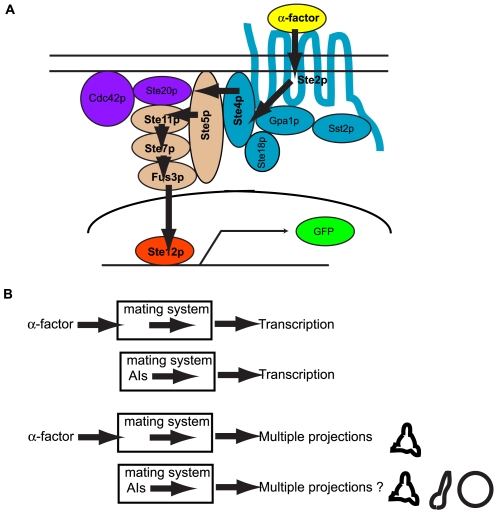
Alternative Inputs to *α*-factor. (A) The α-factor-transcription pathway in the yeast mating signaling network. This signaling pathway contains seven key proteins (Ste2p, Ste4p, Ste5p, Ste11p, Ste7p, Fus3p, and Ste12p) between α-factor and transcriptional activation. An alternative input for each of these components was created. The blue proteins (Ste2p, Ste4p, Gpa1p, Ste18p, Sst2p) belong to the heterotrimeric G-protein cycle, the brown proteins represent the MAPK cascade (Ste5p, Ste11p, Ste7p, Fus3p), the red protein is the transcription factor Ste12p, and the purple proteins (Cdc42p, Ste20p) are involved in cell polarization as well as MAPK signaling. The production of GFP from an integrated *P_FUS1_-GFP* reporter provided the read-out for pheromone-induced transcription. (B) Experimental overview for using alternative inputs to α-factor to investigate cell morphology. When α-factor or alternative inputs to α-factor are added, cells induce transcriptional activation. When α-factor is added, cells produce multiple projections. We addressed the question whether cells produce multiple projections when alternative inputs are used, and how we can manipulate cell morphology using alternative inputs.

**Figure 2:**
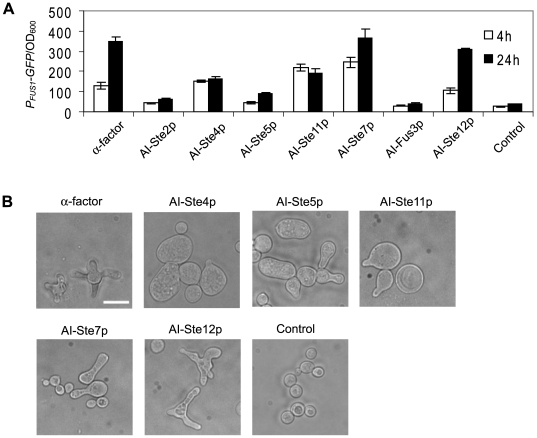
Two different outputs produced by alternative inputs to α-factor. (A) Transcriptional activation induced by alternative inputs. Either α-factor (1 µM) or the alternative inputs were added and transcriptional activation was measured at an early time point (t = 4 hours, white bars) and a late time point (t = 24 hours, black bars) using the *P_FUS1_-GFP* reporter. GFP fluorescence (arbitrary units) was normalized by dividing by the cell density (OD_600_ units). The control was cells unstimulated by α-factor or an alternative input. *P_FUS1_-GFP*/OD_600_ values were averaged from at least three measurements, and bar graphs show mean±SEM. (B) Morphologies induced by α-factor (1 µM) or alternative inputs. Bright field images taken at t = 24 h of a typical set of cells for each AI. The morphologies of AI-Ste2p (*P_GAL1_-STE2^P258L S259L^*) and AI-Fus3p (*P_GAL1_-FUS3^I161L^*) are not shown; they resembled the control cells. The scale bar represents 10 µm.

A natural stimulus activates signaling molecules in a pathway resulting in an output response. We define any genetic manipulation (i.e. overexpressing wild-type or constituitively active forms) that can activate the signaling pathway in lieu of the natural input as “Alternative Inputs or (AIs)”. Here, we set the goal to induce the natural output using alternative inputs. In this study, we constructed alternative inputs to the yeast mating pheromone α-factor in the pathway leading from α-factor to the transcription of pheromone-inducible genes (Figure 1A). The signaling proteins were overexpressed from the *P_GAL1_* promoter on a multi-copy 2μ plasmid. After inducing expression of the alternative input with galactose, we monitored two different outputs, transcriptional activation of the reporter *P_FUS1_-GFP* and cell morphology, at an early (4 hours) and a late time point (24 hours) (Figure 2). We quantified transcription in terms of GFP fluorescence per unit of cell density (*P_FUS1_-GFP*/OD_600_).

In all experiments, the cells contained deletions of the *BAR1* and *MFα1* genes; we refer to the *bar1Δ mfα1Δ* strain background as “wild-type.” *BAR1* encodes for an α-factor protease; *MFα1* encodes for α-factor along with the *MFα2* gene. We deleted *MFα1* because of a concern that a small fraction of cells could switch from *MAT* ***a*** to *MATα* and then synthesize α-factor; *MFα1* is the major source of α-factor in *MATα* cells [10].

We focused on 7 signaling proteins of the α-factor transcription pathway: Ste2p, Ste4p, Ste5p, Ste11p, Ste7p, Fus3p, and Ste12p. First, we attempted to overexpress the wild-type versions of these proteins (Figure S1). Three (Ste4p [11], [12], Ste5p, Ste12p [13]) were able to induce transcription of the *P_FUS1_-GFP* reporter significantly above the basal level, but four did not (Ste2p, Ste11p, Ste7p, Fus3p) (Figure S1A). As a result, we constructed constitutively active forms of Ste2p (Ste2p^P258L, S259L^ [14]), Ste11p (Ste11ΔN, [15]), Ste7p (Ste11ΔN-Ste7p [16]) and Fus3p (Fus3p^I161L^ [17]), and overexpressed them from the *P_GAL1_* promoter on the multi-copy plasmid. Overexpression of Ste2p^P258L, S259L^ and Fus3p^I161L^ weakly induced transcription (Figure 2A), whereas the constitutively active forms of Ste11p and Ste7p activated transcription potently (Figure 2A). Taken together, we had four strong AIs capable of activating transcription to within a factor of two of α-factor (AI-Ste4p, AI-Ste11p, AI-Ste7p, AI-Ste12p), one moderately weak AI (AI-Ste5p), and two weak AIs (AI-Ste2p, AI-Fus3p).

Quite strikingly, the morphologies of the AI strains differed significantly from the morphologies caused by α-factor. Wild-type cells treated with a high concentration of α-factor for an extended period (t = 24 hours) induced multiple projections (Figure 2B) [8]. Only AI-Ste12p induced multiple projections, although as we demonstrated later, this phenotype was caused by the unexpected production of α-factor. Overexpression of AI-Ste2p or AI-Fus3p resulted in negligible morphological changes presumably because of low transcriptional activation. AI-Ste4p, on the other hand, produced large (round) cells (Figure 2B). Overexpression of AI-Ste5p induced an elongated morphology (Figure 2B). Morphologies induced by AI-Ste11p included both large round cells and cells containing a single projection. For AI-Ste7p, most of the responding cells possessed one long projection (90%), and a few cells had a second projection (7%). Overall, there was rough trend from round cells to more polarized cells with each succeeding AI down the pathway.

**Figure 3:**
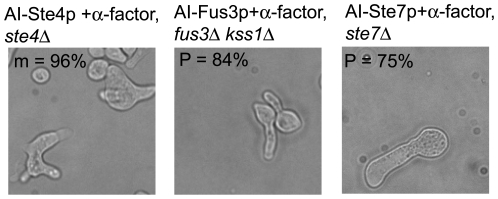
Morphologies induced by AIs + *α*-factor. Bright field images taken at t = 24 h of AI cells induced with galactose and treated with 1 µM α-factor. AI-Ste4p, AI-Ste7p, and AI-Fus3p cells were each exposed to α-factor. To ensure that the responding cells had not lost the AI plasmid, each alternative input was overexpressed in its deletion strain (genotypes are above the images). The percent (%) of the most predominant phenotype (m = multiple projections, i.e. more than three; P = one long projection) is shown at the top-left of each image. At least 100 responding cells were analyzed in each strain.

To further investigate the trend down the pathway from less polarized round cells (AI-Ste4p) to more polarized cells with a single projection (AI-Ste7p), we simultaneously added α-factor with the inducer galactose in AI-Ste4p, AI-Ste7p, and AI-Fus3p cells. Interestingly, we found that AI-Ste4p+α-factor produced cells with multiple projections (96%, Figure 3) suggesting that α-factor was dominant in this combination. On the other hand, the AI-Ste7p+α-factor combination gave rise to cells with a single long projection (75%, Figure 3) similar to AI-Ste7p alone suggesting that the AI was dominant over α-factor in this case. The AI-Fus3p+α-factor combination also gave rise to cells with a single long projection (84%, Figure 3), even though AI-Fus3p alone had no morphology phenotype because of weak transcriptional activation.

### Alternative inputs caused localization defects in polarity markers

**Figure 4:**
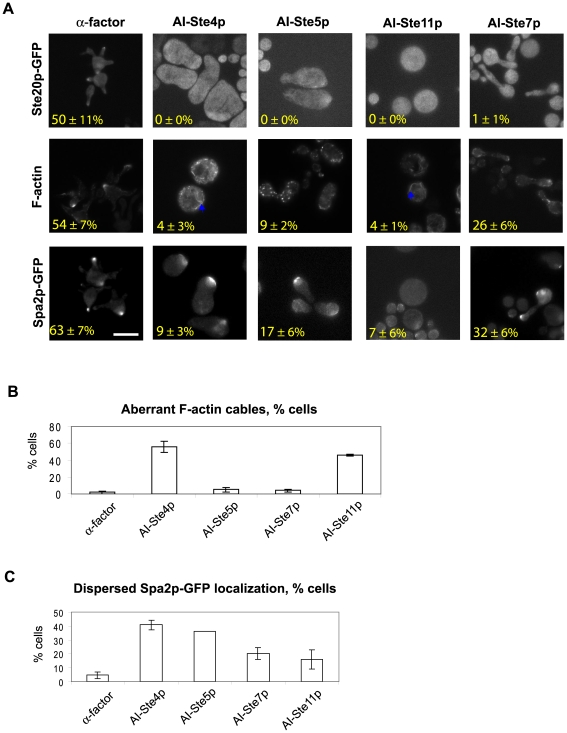
Alternative inputs caused mislocalization of polarity markers. (A) Localization of Ste20p-GFP, F-actin and Spa2p-GFP in cells stimulated by α-factor or selected alternative inputs. The GFP-tagged proteins were integrated into the genome; F-actin was stained with rhodamine-conjugated phalloidin. After 24 hours of induction, the cells were fixed and visualized by fluorescence microscopy. The percent (%) of proper localization is shown at the bottom-left in each figure and represents the percentage of cells exhibiting the canonical localization pattern for the marker when stimulated by α-factor. Note that not all α-factor treated cells showed this pattern. At least three independent experiments were analyzed for each strain. The blue arrows indicate aberrant actin cables. The scale bar represents 10 µm. (B) Percent of cells containing aberrant F-actin cables. Thick disorganized cables were categorized as aberrant. Data is from at least three independent experiments per input (t = 24 h). (C) Percent of cells showing dispersed Spa2p-GFP localization. Most cells treated with α-factor showed a punctuate patch near the tip of the mating projection. Cells induced by the AIs showed Spa2p-GFP distributed more diffusely along the membrane and in the cytoplasm, which was categorized as a dispersed localization pattern. The numbers of cells with this dispersed localization pattern were counted for each input (at least three independent experiments per input).

To perform a more detailed characterization of the morphological changes induced by the alternative inputs, we investigated the localization of three cell polarity markers (Figure 4A) in the four AI strains AI-Ste4p, AI-Ste5p, AI-Ste11p, and AI-Ste7p. Ste20p is a kinase for Ste11p and an effecter of Cdc42p that binds active Cdc42p, serving as an important link between MAPK signaling and cytoskeletal organization [6], [18], [19]. In α-factor treated cells, Ste20p-GFP translocates from the cytoplasm to the membrane of the mating projection. In yeast, the mating response polarizes the two types of filamentous actin (F-actin) structures: patches and cables. The actin patches localize to the mating projection tip, and the actin cables extend from the tip to the interior of the cell [20]. Spa2p is a primary constituent of the polarisome [21], which is involved in actin polymerization, and polarized transport and secretion. In wild-type cells treated with α-factor, Spa2p-GFP localizes at the very tip of the projection as a punctuate patch (Figure 4A).

Compared to cells stimulated with α-factor, AI-activated cells displayed severe defects in the spatial patterns of the polarity markers (Figure 4A). In particular, there was a significant loss in the polarization of Ste20p-GFP. For all 4 AIs, there was a dramatic mislocalization of Ste20p-GFP to the cytoplasm. These results suggest that projection morphologies induced by AI-Ste5p and AI-Ste7p do not require the localization of Ste20p to the projection tip [22].

F-actin and Spa2p had a somewhat more polarized appearance in the AI cells compared to Ste20p. AI-Ste7p had substantial actin patch formation (26%) in the mating projection. On the other hand, AI-Ste4p (56%) and AI-Ste11p (46%) induced aberrant actin cable structures in addition to patch structures (Figure 4B). These cables were thick and disorganized, and found predominantly in the large round cells. For Spa2p-GFP, there was some degree of polarization in all four AIs with AI-Ste7p showing the most proper polarization (32%) followed by AI-Ste5p (17%). However, there was also a new phenotype that was observed in the AI strains and not in the α-factor treated cells: a dispersed distribution of Spa2p-GFP that spread along the membrane and also into the cytoplasm. AI-Ste4p showed the highest level of dispersed Spa2p followed by AI-Ste5p. AI-Ste11p and AI-Ste7p showed lower levels of dispersed Spa2p (Figure 4C). Taken together, AI-Ste7p and to a lesser extent AI-Ste5p showed a moderate level of polarization for F-actin and Spa2p, but not for Ste20p. AI-Ste4p and AI-Ste11p showed poor polarization for all the markers.

### Morphology AIs-Deletions matrix

**Figure 5:**
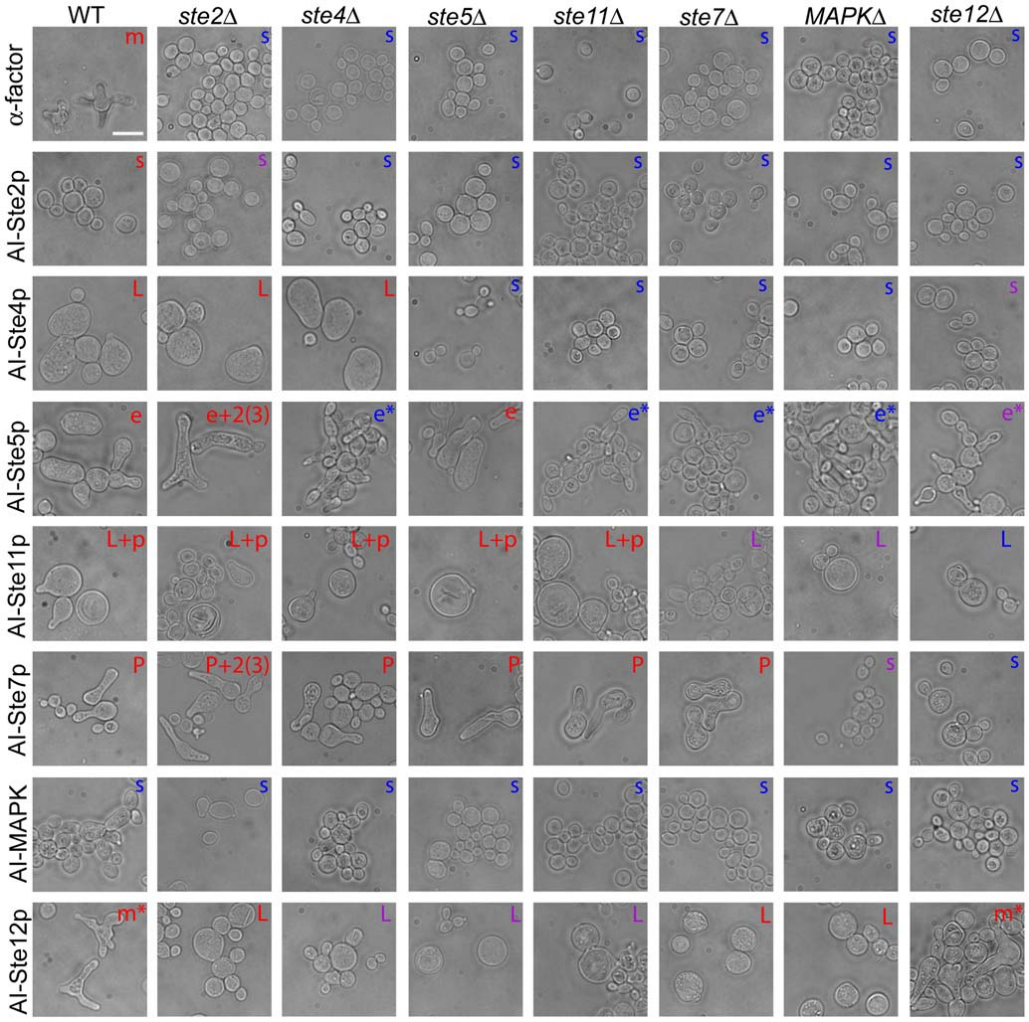
AIs-Deletions matrix of morphology. Bright field images of cells containing all possible combinations of inputs and deletions were taken after 24 hours and the observed morphologies were classified (details in materials and methods). The characters located in the top-right corner of each picture indicate the representative morphological phenotype. The classification scheme is as follows: (a) m = multiple projections (more than three); (b) m* = multiple projections that depend on the *MFα2* gene; (c) 2(3) = two or three projections; (d) P = one long projection; (e) p = one short projection; (f) e = elongated cells including cells with one projection; (g) e* = elongated cells including budding cells; (h) L = large cells; (i) s = small cells. The color of the characters indicate the degree of transcriptional activation: *P_FUS1_-GFP*/OD_600_ <50 (blue); 50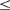 *P_FUS1_-GFP*/OD_600_ <60 (purple); *P_FUS1_-GFP*/OD_600_ 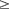
60 (red). The scale bar represents 10 µm.

In wild-type cells, no single alternative input in the α-factor-transcription pathway was able to induce multiple (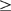
3) projections, although four AIs (AI-Ste4p, AI-Ste11p, AI-Ste7p, and AI-Ste12p) possessed strong transcriptional activation. To characterize the morphologies induced by the AIs more systematically and to search for new morphologies, we combined the gain-of-function alternative inputs with loss-of-function deletions. We constructed all combinations of alternative inputs and deletions among the 7 signaling genes and the resulting phenotypes were summarized in two AIs-Deletions matrices, one for transcriptional activation (Table 1) and one for morphology (Figure 5). Here, the convention is that the rows contain natural input (first row) followed by the different AIs, and the columns contain the wild-type background (first column) followed by the different deletions. In the morphology AIs-Deletions matrix, there were two combinations that produced multiple projections: AI-Ste5p *ste2Δ* and AI-Ste7p *ste2Δ* (Figure 5). In addition, we found several interesting results among the other entries of the morphology matrix.

**Table 1:** AIs-Deletions matrix of transcriptional activation.

*P_FUS1_-GFP*/OD_600_ (t = 24 h)*								
Input	WT	*ste2Δ*	*ste4Δ*	*ste5Δ*	*ste11Δ*	*ste7Δ*	*MAPKΔ*	*ste12Δ*
**α** ***-*** **factor**	**350**	**33**	**32**	**41**	**35**	**36**	**34**	**38**
**AI-Ste2p**	**61**	**51**	**38**	**41**	**39**	**37**	**37**	**37**
**AI-Ste4p**	**164**	**234**	**182**	**46**	**44**	**40**	**42**	**54**
**AI-Ste5p**	**87**	**129**	**42**	**83**	**45**	**45**	**47**	**59**
**AI-Ste11p**	**190**	**151**	**145**	**170**	**163**	**53**	**59**	**42**
**AI-Ste7p**	**365**	**265**	**213**	**310**	**280**	**289**	**54**	**41**
**AI-MAPK**	**41**	**46**	**35**	**41**	**37**	**36**	**46**	**39**
**AI-Ste12p**	**310**	**107**	**54**	**50**	**59**	**64**	**70**	**84**

* *P_FUS1_-GFP*/OD_600_ values were averaged from at least three measurements.

We classified the output into different morphological classes based on representative cells from each combination. The categories included multiple projection cells (m), single long projection cells (P), single short projection cells (p), elongated cells (e), large cells (L), and small round cells (s). As we expected, the most general trend was that morphology was influenced by transcriptional activation (Table 1) so that in general the elements above the matrix diagonal showed the small round morphology (Figure 5). For example, the large cells induced by AI-Ste4p were observed in the wild-type, *ste2Δ*, and *ste4Δ* strains, but not observed in the *ste5Δ*, *ste11Δ*, *ste7Δ*, *MAPKΔ*, and *ste12Δ* strains, and the single long projection induced by AI-Ste7p was observed in any deletions strain of upstream of MAPK, but not observed in the *MAPKΔ*, and *ste12Δ* strains. There were some exceptions to this general trend, however, as we describe below.

Interestingly, AI-Ste5p induced polarized phenotypes (i.e. elongated cells) in all strains including deletions downstream of *STE5* in the α-factor transcription pathway. In the absence of transcriptional activation, AI-Ste5p produced elongated cells and elongated cells that formed a bud or another elongated cell (Figure 5). These morphologies were clearly distinct from unstimulated cells undergoing vegetative budding. Presumably, the budding in the AI-Ste5p cells occurred because of imperfect cell-cycle arrest caused by the low levels of MAPK signaling, which was blocked by the downstream deletions. These data demonstrate that Ste5p possesses a polarizing function that is independent of MAPK signaling and pheromone-induced transcription.

AI-Ste11p induced both large round cells and cells with a projection in the deletions upstream and including *STE11*, but in deletions downstream of *STE11*, it induced only large round cells (Figure 5). Ste11p can activate at least three different pathways including the mating pathway (the α-factor transcription pathway), the invasive growth pathway, and the HOG pathway. In previous work [16], Harris et al. have demonstrated that large round cells could arise from induction of the HOG pathway by Ste11p, and our data is consistent with this view. We hypothesize that activation of the HOG pathway was responsible for the round cells and activation of the mating pathway gave rise to the polarized cells containing a projection.

AI-Ste12p induced large round cells in any deletion strain except for the *ste12Δ* strain. The multiple projections phenotype in the wild-type backgrounds was the result of the production of α-factor from the *MFα2* gene (Text S1 and Figure S2). In the other deletions, α-factor signaling was blocked giving rise to morphologies and transcriptional activation comparable to AI-Ste12p in the *mfα2Δ* strain (Table 1, Figure 5 and S2). Control experiments with the other AIs showed no differences caused by the absence of *MFα2* (data not shown). AI-Ste4p, AI-Ste11p, and AI-Ste12p (*mfα2Δ*) all formed large round cells. One hypothesis is that the large round phenotype was caused by transcriptional activation (either pheromone or HOG) in the absence of polarization. Thus, the results in this section highlight cell morphology as a highly informative output.

### Multiple projections induced by Alternative Inputs without α-factor

**Figure 6:**
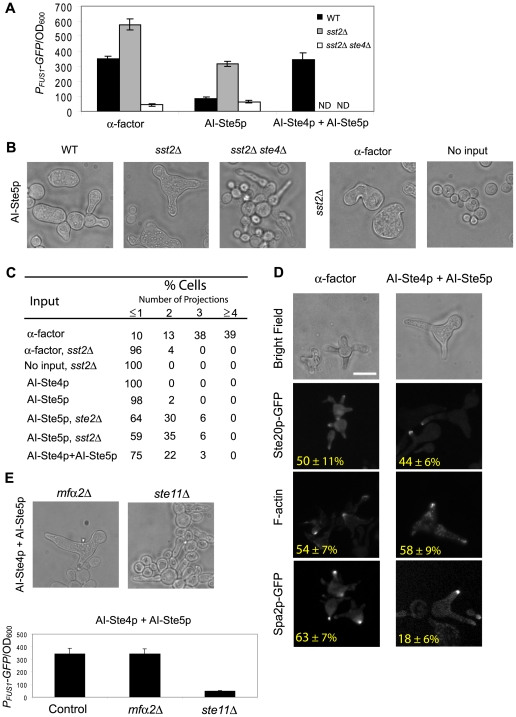
Multiple projections induced by alternative inputs without α-factor. (A) Transcriptional activation of *P_FUS1_-GFP* (t = 24 h) by 1 µM α-factor, AI-Ste5p, and AI-Ste4p + AI-Ste5p. For the first two inputs, the strain backgrounds were wild-type (black), *sst2Δ* (gray), and *sst2Δ ste4Δ* (white); AI-Ste4p + AI-Ste5p was measured only in the wild-type background and not determined (ND) in *sst2Δ* and *sst2Δ ste4Δ* strains. *P_FUS1_-GFP*/OD_600_ values were averaged from at least three measurements, and bar graphs show mean±SEM. (B) Morphologies of AI-Ste5p-stimulated cells in wild-type, *sst2Δ*, and *sst2Δ ste4Δ* strain backgrounds (t = 24 h). Morphologies of *a*-factor (1 µM) and non-stimulated cells in an *sst2Δ* strain background (t = 24 h). (C) Numbers of projections produced by AI-Ste5p and (AI-Ste4p + AI-Ste5p) cells. For each of the inputs and strain backgrounds, we determined the percent of cells with 0 or 1, 2, 3, and 4 or greater projections (t = 24 h, at least 100 responding cells). (D) Localization of polarity markers in (AI-Ste4p + AI-Ste5p)-induced cells. The percent (%) of proper localization is shown and was determined as described previously in Figure 4A (at least 100 responding cells with more than one projection were counted). Images of wild-type cells treated with α-factor are reproduced from Figure 4A. Scale bar = 10 µm. (E) Morphologies and transcriptional activation of (AI-Ste4p + AI-Ste5p) cells in *mfα2Δ* and *ste11Δ* strain backgrounds (t = 24 h). *P_FUS1_-GFP*/OD_600_ values were averaged from at least three measurements, and bar graphs show mean±SEM.

In the AIs-Deletions matrix, there were two combinations that produced multiple projections: AI-Ste5p *ste2Δ* and AI-Ste7p *ste2Δ* (Figure 5). We chose to focus on the former because AI-Ste5p *ste2Δ* produced more 2^nd^ and 3^rd^ projections, and because the difference between AI-Ste5p *ste2Δ* versus AI-Ste5p alone (Figure 6C) was more dramatic than AI-Ste7p *ste2Δ* versus AI-Ste7p alone. In addition to the morphological difference, the AI-Ste5p *ste2Δ* strain also exhibited significantly greater pheromone-induced transcription than AI-Ste5p in the wild-type background (Table 1). We hypothesized that this phenotype was caused by the loss of Sst2p activity rather than the loss of receptor Ste2p function; Sst2p is an RGS (Regulator of G-protein Signaling) protein that catalyzes the deactivation of heterotrimeric G-protein [23]. Recently, Ballon et. al. demonstrated that Ste2p tethers Sst2p to the membrane through the DEP domain of Sst2p [24], and that the absence of Ste2p will cause Sst2p to be localized exclusively to the cytoplasm. To test this hypothesis, we overexpressed AI-Ste5p in an *sst2Δ* strain background. The morphological patterns were almost identical between AI-Ste5p *ste2Δ* and AI-Ste5p *sst2Δ* (Figure 6C), suggesting that the ability to form multiple projections in AI-Ste5p *ste2Δ* was because of elevated basal heterotrimeric G-protein activity [25], which also led to an enhanced transcriptional response.

To investigate whether these increased mating responses were mediated by Gβγ, the *STE4* gene was deleted along with the *SST2* gene, and AI-Ste5p was overexpressed. The transcriptional activity and morphological changes induced by AI-Ste5p in the *sst2Δ* strain were completely eliminated in the *sst2Δ ste4Δ* background (Figure 6A and 6B), demonstrating that Ste4p was necessary for the morphological gain-of-function of cells with AI-Ste5p in the *sst2Δ* strain. We cannot rule out a possible role for Gpa1p (Gα) because the basal activity of both Gβγ and activated Gpa1p would presumably increase in the *ste2Δ* and *sst2Δ* strains.

To test whether Ste4p was also sufficient in combination with Ste5p to induce multiple projections, we simultaneously overexpressed both AI-Ste5p and AI-Ste4p in the wild-type background. Indeed, the double AI strain contained cells with two and three projections, whereas each individual alternative input induced zero or one projection (Figure 6C). As a control, we also observed this gain-of-function phenotype in the *mfα2Δ* background (Figure 6D). Deleting *STE11* resulted in a loss of transcriptional activation and the absence of multiple projections (Figure 6D).

In addition, overexpression of both AI-Ste4p and AI-Ste5p corrected many of the localization defects in the polarity markers observed when AI-Ste4p and AI-Ste5p were applied singly (Figure 6E). Indeed, the localization of two of the markers was quite similar to α-factor treated cells in contrast to the individual AIs which showed dramatic disruption. For example, AI-Ste4 and AI-Ste5 each exhibited 0% of cells with Ste20p-GFP polarized, and instead Ste20p-GFP was almost exclusively cytoplasmic. By contrast, 44% of AI-Ste4+AI-Ste5 cells that formed more than one projection contained Ste20p-GFP polarized near the tip of the mating projection with little cytoplasmic staining. The one marker that was not localized properly in the (AI-Ste4p+AI-Ste5p) cells was Spa2p-GFP (18%). The low percentage was the result of diminished Spa2p-GFP fluorescence rather than the dispersed localization pattern observed in AI-Ste4p cells or AI-Ste5p cells. We note that a *spa2Δ* strain exposed to α-factor can still make multiple projections although with altered morphology and timing [8]. Thus, these data provided evidence for synergy between Ste4p and Ste5p on cell morphology, for a correlation between making multiple projections and the proper spatial dynamics of the polarity markers Ste20p and the actin patches, and for the existence of additional factors other than Ste4p and Ste5p that may be necessary for Spa2p localization.

### Membrane targeting of Ste5p promotes formation of more than one projection

**Figure 7:**
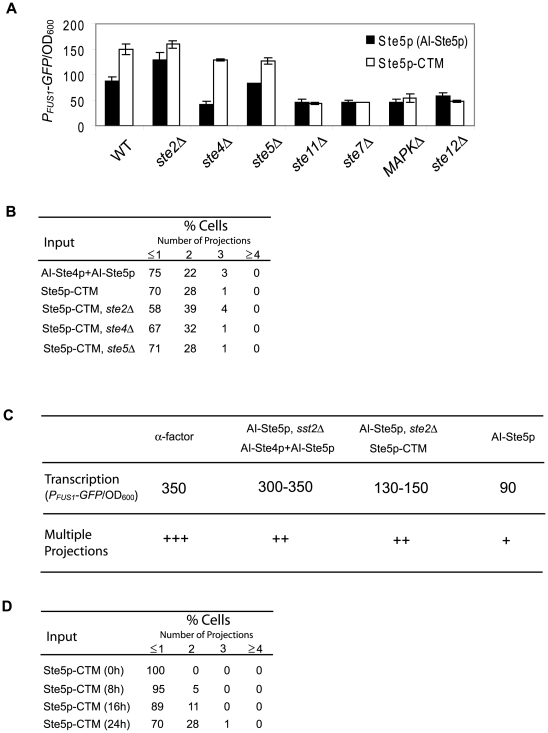
Multiple projections induced by membrane targeting of Ste5p. (A) Transcriptional activation induced by Ste5p-CTM. Ste5p (AI-Ste5p, black) and Ste5p-CTM (white) were overexpressed in a wild-type strain and the seven deletion strains of the mating pathway. Transcriptional activation was measured at t = 24 h. *P_FUS1_-GFP*/OD_600_ values were averaged from at least three measurements, and bar graphs show mean±SEM. (B) Numbers of projections produced by AI-Ste4p+AI-Ste5p and Ste5p-CTM cells in a wild-type background and Ste5p-CTM cells in *ste2Δ*, *ste4Δ*, and *ste5Δ* strains. For each of the inputs and strain backgrounds, we determined the percent of cells with 0 or 1, 2, 3, and 4 or greater projections (t = 24 h, at least 100 responding cells). (C) Correlation between transcriptional activation and numbers of projections in Ste5p strains. Transcriptional activation (*P_FUS1_-GFP*/OD_600_ values) and numbers of multiple projections (“+++” indicates WT levels of projections, “++” indicates more projections than AI-Ste5p (indicated as “+”) but fewer projections than WT) were summarized for each genetic manipulation with Ste5p that produced multiple projections. (D) Time-course of number of projections produced by Ste5p-CTM. For each time point, we determined the percent of cells with 0 or 1, 2, 3, and 4 or greater projections (t = 8, 16, 24 h, at least 100 responding cells). At t = 0 h, we observed more than 400 cells, and responding cells were less than 1%.

Ste4p recruits Ste5p to the plasma membrane in response to α-factor, and forced membrane targeting of Ste5p using a C-terminal membrane tag (Ste5p-CTM) activates the MAPK cascade without Ste4p [3]. We hypothesized that in the (AI-Ste4p+AI-Ste5p) cells Ste4p performed the role of recruiting Ste5p to the plasma membrane. To test this hypothesis, we overexpressed Ste5p-CTM instead of Ste5p. Indeed, Ste5p-CTM enhanced the transcriptional response and produced more second projections even in the complete absence of Ste4p (Figures 7A and 7B).

These data suggest that a minimum level of transcriptional activation is necessary to form multiple projections. AI-Ste5p possessed a low level of transcriptional activation (*P_FUS1_-GFP*/OD600 = 87), and increasing transcription (130 to 150) in the Ste5p-CTM and AI-Ste5p *ste2Δ* strains resulted in multiple projections (Figure 7C). However, the correlation between transcription levels and the ability to make multiple projections is somewhat loose. AI-Ste5p *sst2Δ* cells and (AI-Ste4p+AI-Ste5p) cells possess mating transcriptional activity close to wild-type cells treated with α-factor, and yet they make two projection instead of three (Figure 7C). Finally, we note that *sst2Δ* cells treated with α-factor showed dramatically stronger transcriptional activation than wild-type cells treated with α-factor, but that the *sst2Δ* cells formed only a single projection (Figure 6A and 6B, [9]). Thus, too much or too little pheromone-induced transcription may be incompatible with making multiple projections, suggesting that an intermediate amount of transcriptional activation is important for multiple projections formation.

Certain genetic manipulations can lead to simultaneous formation of multiple sites of polarization (i.e. polar caps) [26]. On the other hand, the formation of multiple projections induced by α-factor is sequential [8]. It is important to distinguish whether the multiple projections induced by alternative inputs were formed sequentially or simultaneously. We performed time-course experiments (t = 0, 8, 16, 24 hours) in Ste5p-CTM cells that produced multiple projections (Figure 7D), and monitored how the second projections were produced. At 8 hours after galactose treatment, most of the cells produced only a single projection, whereas at t = 16 hours, 11% of cells produced a second projection. At t = 24 hours, 28% of cells produced a second projection and 1% of cells produced a third projection. These results suggest that Ste5p-CTM induced the second projection not simultaneously but sequentially although we cannot rule out the possibility that the first projection did not stop growing after the second projection was initiated from these time-course experiments. Preliminary time-lapse studies with GFP-Ste5p-CTM indicated that the first projection stops before the start of the second projection (T.-M. Yi, data not shown).

### Effects of varying the level of alternative inputs on transcription and morphology

**Figure 8:**
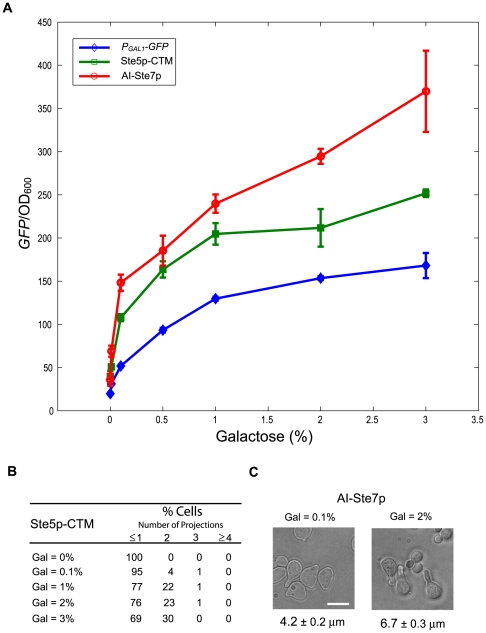
Transcription and morphology as the level of alternative inputs is varied. (A) Transcriptional activation induced by Ste5p-CTM and AI-Ste7p. Ste5p-CTM (green square) and AI-Ste7p (red circle) were overexpressed in a *gal2Δ* strain. Induction level of alternative inputs was estimated using *P_GAL1_-GFP* reporter (blue diamond). Transcriptional activation was measured at t = 24 h. *P_FUS1_-GFP*/OD_600_ values were averaged from at least three measurements, and error bars show mean±SEM. (B) Numbers of projections produced by Ste5p-CTM in a *gal2Δ* strain background. For each galactose concentration, we determined the percent of cells with 0 or 1, 2, 3, and 4 or greater projections (t = 24 h, Gal = 0.1, 1, 2, 3%, at least 100 responding cells). At Gal = 0%, we observed more than 400 cells, and responding cells were less than 1%. (C) Morphological phenotypes produced by AI-Ste7p in a *gal2Δ* strain background. Morphologies at Gal = 0.1% (left) and at Gal = 2% (right). The average projection length (measured from 50 cells) is shown below each picture. Scale bar = 10 µm.

It is instructive to investigate the outputs in response to varying the level of alternative inputs. To this end, we created *gal2Δ* strains [27], which allows a more graded activation of the *P_GAL1_* promoter by galactose, and treated the Ste5p-CTM and AI-Ste7p strains with several concentrations of galactose (Figure 8). We were interested in the correlation between transcription and morphology (e.g. number of projections). In both strains, transcriptional activation showed a graded response from 0.1% to 1% galactose. In AI-Ste7p cells, there was also a graded response from 1% to 3% galactose, whereas AI-Ste5p-CTM cells showed more of a saturated response in this range. When transcriptional activation was around 100 (*P_FUS1_-GFP*/OD600, 0.1% galactose) in Ste5p-CTM, most cells had only one projection (Figure 8B), and this result was consistent with the table in Figure 7C, which suggests that a minimum level of transcriptional activation was necessary for making more than one projection. We also observed an intermediate phenotype in AI-Ste7p cells in which cells had a shorter projection (4.2±0.2 µm) when transcriptional activation was around 150 (0.1% galactose) compared to cells that had a higher induction level (6.7±0.3 µm, 2% galactose, Figure 8C). Taken together, these results suggest that there is some correlation between transcriptional activation and morphology, but the relationship is complex. In AI-Ste7p, greater transcriptional activation resulted in a longer projection but only a single projection is made; in Ste5p-CTM, greater transcriptional activation resulted in more projections. Clearly, there is a fundamental difference between the AI-Ste7p and the Ste5p-CTM strains.

## Discussion

### Synthetic morphology using alternative inputs

In this study, we attempted to reproduce in the absence of mating pheromone the multiple mating projections phenotype of yeast cells. We applied a novel synthetic approach termed “Alternative Inputs” to this problem. Whereas wild-type cells exposed continuously to α-factor form multiple mating projections, we found that none of the AIs alone could induce multiple projections.

During the course of this study, we identified genetic combinations that could produce multiple projections: (1) AI-Ste5p *ste2Δ*, (2) AI-Ste7p *ste2Δ*, (3) AI-Ste5p *sst2Δ*, (3) AI-Ste5p+AI-Ste4p, and (4) Ste5p-CTM. As we describe below, these results shed light on this morphology, as well as highlight the differences between making one projection versus making more than one projection. Thus, we re-engineered the multiple projections mating morphology using alternative inputs without α-factor.

### Morphologies induced by pheromone

We attempted to recapitulate the multiple projections phenotype induced by high concentrations of α-factor (1 µM). It is important to note the effect of pheromone dose on the morphology of mating projections, which has been reported in the literature. Dose response curves for α-factor induced projection formation were measured, as well as cell division arrest and agglutination [28]. Recent studies using microfluidics devices showed that the shape of the projection(s) ranged from wide projections (lower concentrations, e.g. 10 to 40 nM) to thin projections (higher concentrations, e.g. 100 to 1000 nM) depending on pheromone levels, and that double projections at 6 hours were observed at higher α-factor levels but not at lower concentrations [29].

In most cases, multiple projections induced by high concentrations of α-factor are formed by a succession of polarized growth at new sites and not by simultaneous growth at several sites. The multiple projections formation presumably requires oscillations either in protein levels, activities, or localization in the cell [9]. One may be concerned that such oscillations might thus be precluded by over-expression of a protein (whose transcriptional level could then not be regulated anymore), or by expression of a constitutively active form of the protein. Indeed, when both α-factor and alternative inputs were added, AI-Ste7p and AI-Fus3p (both are constitutively active forms) were dominant to α-factor although α-factor was dominant to AI-Ste4p (a wild-type form, Figure 3). However, it is noteworthy that AI-Ste5p *ste2Δ*, AI-Ste5p *sst2Δ*, (AI-Ste4p+AI-Ste5p) cells (proteins levels are not controlled by α-factor, but by the *GAL1* promoter) and even Ste5p-CTM (a constitutively active form of Ste5p) cells produced multiple projections. These data argue that other parts of the network may overcome the loss of regulation of a specific component.

### Morphologies induced by single alternative inputs

No single alternative input could induce multiple projections (Figure 2B); instead we observed a variety of morphologies ranging from round to elongated to single projection cells. Surprisingly, AI-Ste5p could induce a polarized phenotype even in the absence of MAPK signaling and transcriptional activation. One hypothesis to explain this finding is that Ste5p is an early marker of polarization that is sensitive to internal polarity cues [30]. Once on the membrane, it can serve as a scaffold for polarization, and this function does not depend on an active MAPK cascade. Ste4p, on the other hand, when overexpressed might not efficiently localize at the internal cue. Indeed, Ste18p-GFP (Gγ), an indirect marker of Ste4p (Gβ) localization, is broadly distributed at the plasma membrane in AI-Ste4p cells (Tanaka and Yi, unpublished data), perhaps contributing to the round phenotype of AI-Ste4p cells.

AI-Ste7p produced a single projection and induced high transcriptional activation comparable to transcription induced by α-factor in wild-type cells (Figure 2A). However, AI-Ste7p cells did not make more than one projection, and this single-projection phenotype was dominant even in the presence of α-factor. These data suggest that MAPK signaling may be part of a positive feedback loop which when sufficiently sustained results in a single projection that does not terminate.

Interestingly, the individual AIs all showed significant defects in the localization of polarity markers Ste20p, F-actin, and Spa2p. Thus, proper localization of these proteins is not required for making a single projection. In the case of Ste20p, Peter and colleagues showed that the Ste20p mutant lacking the entire CRIB domain that cannot bind Cdc42p was able to fully activate the mating MAP kinase pathway and form a single projection although the Ste20p mutant did not localize at the projection [22], and our observations are consistent with this finding.

Previous studies have investigated abnormal mating morphologies arising from genetic perturbations. In particular, Chenevert, Valtz and Herskowitz classified a large number of mutants involved in pheromone-induced cell polarization [31]. They grouped these mutations into three morphological classes: (1) “Shmooless mutants” including mutations in *BEM1* and *CDC24*, which are necessary to establish polarity, (2) “Peanut shmoo mutants” including mutations in *SPA2* and *PEA2*, that result in wide projections, and (3) “Tiny shmoo mutants” including mutations in *TNY1* that produce tiny projections. Most of these mutants resulted from loss-of-function perturbations; it would be informative to compare and contrast gain-of-function morphological phenotypes arising from alternative inputs with these loss-of-function phenotypes. This combined approach may help to further characterize genes that display complex morphological phenotypes (e.g. bending projections) such as *AFR1* [32], [33], which influences septin dyamics.

### Role of Ste5p in making multiple projections

**Figure 9:**
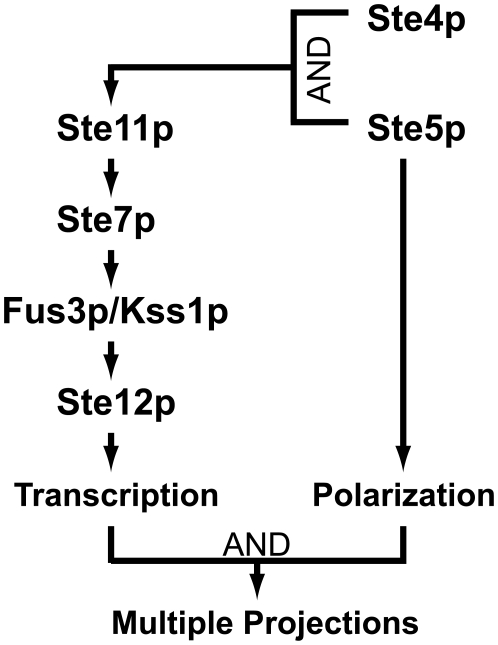
Model for how multiple projections were induced by AI-Ste4p and AI-Ste5p. Arrow diagram explaining multiple projections in (AI-Ste4p + AI-Ste5p) cells. The diagram combines the α-factor transcription pathway with the ability of Ste5p to make projections in the absence of transcriptional activation. Transcription without polarization cannot produce multiple projections, and polarization without transcription likewise does not result in multiple projections. However, the two together at proper strength can make multiple projections.

This research implicates Ste5p as a key player in the formation of multiple projections. Having sufficient transcriptional activation is also important; AI-Ste5p alone could not make multiple projections and possessed a low level of mating transcription. Overexpressing Ste4p+Ste5p produced multiple mating projections, whereas overexpressing Ste4p and Ste5p individually failed to produce them (Figure 2B and 6E). We interpreted these results with a model in which Ste5p possesses a polarizing function as an early marker (described in the previous section) that in combination with Ste4p can give rise to multiple projections; one role Ste4p may play is inducing the appropriate level of transcription (Figure 9). Cells overexpressing Ste5p-CTM even in the absence of Ste4p produced almost as many projections as the (AI-Ste4p+AI-Ste5p) cells (Figure 7A and 7B). These data argue that recruiting Ste5p to the membrane is important and that normally Ste4p is involved in this process. Finally, the (AI-Ste4+AI-Ste5) cells also quite strikingly showed proper localization of two of the three polarity markers suggesting that proper spatial patterning of Ste20p and actin patches are important for multiple projections formation.

Are the oscillatory dynamics that underlie multiple mating projections formation a systems-level property or the outcome of the actions of a single or small set of genes? We believe the former is true, and thus Ste5p is an important player in a complex process. The fact that none of the artificially induced phenotypes completely matches the number of projections produced by α-factor argues that there are additional dynamics and interactions to be investigated.

### A working model explaining morphological phenotypes in terms of the spatial-temporal dynamics of mating pathway component*s*

Our hypothesis is that the spatial-temporal oscillatory proteins dynamics are necessary for forming multiple projections. We propose the following working model based on our data. Intermediate levels of transcriptional activation (130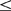 *P_FUS1_-GFP*/OD_600_<350, Figure 7C) are important to induce the synthesis of negative regulators that stop progression of the first projection and reset the cell before the second projection begins. Low transcriptional induction may be able to trigger formation of a mating projection but not sufficient to induce this transcriptional negative feedback resulting in only a single projection, e.g. AI-Ste5p (Figures 2 and 7C). On the other hand, constitutive activation of Fus3p via unregulated MAPK signaling (indicated by high *P_FUS1_-GFP* levels) can create a very strong first projection that cannot be stopped by the negative feedback, which may be partially disabled. More specifically, we hypothesize that persistently activated Fus3p which in turn activates Bni1 [34] can stimulate a positive feedback loop involving the polarized synthesis and transport of mating pathway components [26]. The dominance of the single projection phenotype of AI-Ste7p over α-factor treatment, the single projection phenotype of (AI-Fus3p+αf), and the fact that *sst2Δ* cells treated with α-factor make only a single projection are evidence for this hypothesis. In addition, we interpreted the large (round) cells induced by AI-Ste4p, AI-Ste11p, and AI-Ste12p (Figures 2B and 5) as arising from undirected, isotropic synthesis, transport, and localization of mating polarity proteins. For example, in the case of Ste4p, the protein is uniformly distributed on the cell membrane in these strains (Figure 4A) indicating a disruption of the polarizing positive feedback mechanisms. Extensive quantitative exploration in the future is necessary to test this qualitative working model.

### Comparison to other approaches

There have been several large-scale genetic approaches for dissecting biological systems including single deletion libraries [35], double deletion (synthetic lethal) libraries [36], [37], overexpression libraries [38], and using overexpression to test the robustness of a system [39]. “Alternative Inputs” combines gain-of-function (overexpression) and loss-of-function (deletion) perturbations, and hence is closest in spirit to synthetic dosage lethality analysis [38], [40] in which a reference gene is overexpressed in mutant strains containing potential target mutations. There are several differences in the two approaches, however. First, alternative inputs are defined as overexpressing active signaling molecules that can turn on the pathway rather than just overexpressing the wild-type gene product. Second, the AIs-Deletions matrix describes all possible combinations of alternative inputs and deletions, and not only selected reference genes and target mutations. Third, the AIs approach can be applied to any pathway (e.g. signaling systems) with inputs and outputs so that cell viability is one of many possible read-outs. The alternative inputs approach extends to encompass individual AIs, AIs and deletions, combinations of AIs, and different outputs. Ultimately, one goal is to reproduce the complex behaviors elicited by the natural input by using the coordinated actions of AIs and other perturbations, thereby demonstrating sufficient understanding to re-engineer the system (i.e. synthetic biology) [1], [41].

### Expanding the scope of the “Alternative Inputs” approach

One shortcoming of this work was that we were unable to construct an adequate AI-MAPK; overexpression of Fus3p, Kss1p, and Fus3p^I161L^ all failed to activate transcription above the basal level. Interestingly, however, overexpression of Fus3p^I161L^ with α-factor produced the same phenotype as AI-Ste7p plus α-factor: a single long projection instead of multiple projections (Figure 3). In addition, Fus3p^I161L^ cells possessed a larger halo in a halo assay than wild type Fus3p cells indicating greater sensitivity to α-factor as previously described [17]. These results suggest that Fus3p^I161L^ is indeed a hyperactive mutant, but that it is not sufficiently active in the absence of α-factor to serve as an alternative input in this system. More generally, designing functional alternative inputs for every gene of interest will be a challenge.

We used the *P_GAL1_* promoter on a multi-copy 2μ plasmid to induce alternative inputs; this approach should be easy to scale up. On the downside, there was likely to be cell-to-cell heterogeneity in the levels of the AIs because of variations in plasmid copy number for the expression vector. To address this issue, we constructed an AI-Ste5p strain by integrating the *P_GAL1_-STE5*. Transcriptional activation was weaker than in cells containing the multi-copy plasmid (*P_FUS1_-GFP*/OD_600_ = 63±7 versus 87±9), and the resulting morphological changes were more modest (reduced polarization). These results suggest that the expression level of Ste5p is important to induce the polarized phenotypes for this AI. Thus, one benefit of using the *P_GAL1_* promoter on a multi-copy 2μ plasmid was higher levels of expression.

In the future, we plan to apply the alternative inputs approach on a larger scale to the yeast mating system, as well as to other signaling networks. The broader scope would necessitate improvements in constructing the AIs and strains, output read-outs, data analysis (e.g. automated image analysis using programs such as CalMorph [42] and CellProfiler [43]), and computational modeling.

## Materials and Methods

### Strains and plasmids

Standard genetic techniques were performed according to [44]. Yeast strains and plasmids used in this study are listed in Table 2 and 3, respectively.

**Table 2:** Yeast strains used in this study.

Strain	Genotype	Source
RJD360	*MAT* ***a*** * can1-100 leu2-3-112 his3-11-15 trp1-1 ura3-1 ade2-1*	Ray Deshaies
RJD863	RJD360 *bar1Δ::hisG*	Ray Deshaies
HTY064	RJD863 *mfα1Δ::LEU2 his3Δ::HIS3MX6-P_FUS1_-GFP*	This study
HTY069	RJD863 *SPA2::SPA2-GFP- HIS3MX6*	This study
HTY073	RJD863 *STE20::STE20-GFP- HIS3MX6*	This study
HTY091	RJD863 *mfα1Δ::LEU2*	This study
HTY116	HTY064 *sst2Δ::HYGB*	This study
HTY136	HTY064 *ste2Δ:: KanMX4*	This study
HTY138	HTY064 *ste4Δ:: KanMX4*	This study
HTY146	HTY064 *mfα2Δ::HYGB*	This study
HTY152	HTY064 *fus3Δ:: KanMX4 kss1Δ::HYGB*	This study
HTY158	HTY064 *ste7Δ:: KanMX4*	This study
HTY159	HTY064 *ste11Δ:: KanMX4*	This study
HTY160	HTY064 *ste12Δ::HYGB*	This study
HTY162	HTY138 *sst2Δ:: ura3Δ58*	This study
HTY167	HTY064 *ste5Δ::HYGB*	This study
HTY175	HTY064 *gal2Δ::HYGB*	This study
HTY176	RJD863 *gal2Δ::HYGB*	This study

**Table 3:** Plasmids used in this study.

Name	Description	Vector base	Source
pHT001	2μ *URA3 P_GAL1_-STE2*	pYES2	This study
pHT002	2μ *URA3 P_GAL1_-STE2(P258L/S259L)*	pYES2	This study
pHT003	2μ *URA3 P_GAL1_-STE4*	pYES2	This study
pHT004	2μ *URA3 P_GAL1_-STE5*	pYES2	This study
pHT005	2μ *URA3 P_GAL1_-STE11*	pYES2	This study
pHT006	2μ *URA3 P_GAL1_-STE11ΔN*	pYES2	This study
pHT007	2μ *URA3 P_GAL1_-STE7*	pYES2	This study
pHT008	2μ *URA3 P_GAL1_-STE11ΔN-STE7*	pYES2	This study
pHT009	2μ *URA3 P_GAL1_-FUS3*	pYES2	This study
pHT010	2μ *URA3 P_GAL1_-FUS3(I161L)*	pYES2	This study
pHT011	2μ *URA3 P_GAL1_-KSS1*	pYES2	This study
pHT012	2μ *URA3 P_GAL1_-STE12*	pYES2	This study
pHT013	2μ *URA3 P_GAL1_-STE5-CTM*	pYES2	This study
pHT014	2μ *TRP1 P_GAL1_-STE4*	pYES3/CT	This study
pHT015	2μ *URA3 P_GAL1_-GFP*	pYES2	This study

The *P_FUS1_-GFP* reporter (*HIS5*-marked PCR fragment) [45] was targeted to the *HIS3* locus of the strain RJD863 by PCR-based gene integration to create the strain HTY028. Then, the *mfα1Δ* strain HTY064 was constructed by PCR-based gene disruption of HTY028. In this study, HTY064 was used as the “wild-type” strain in most experiments, and all deletion strains were derived from HTY064 by PCR-based gene disruption.

The strains containing the GFP-tagged polarity markers were constructed by the C-terminal integration of GFP (*HIS5*-marked PCR fragment). GFP was fused to the C-terminus of the *SPA2* gene (HTY069) and the *STE20* gene (HTY073) in the strain RJD863. To construct Ste18p-GFP, GFP was inserted directly in front of the prenylation consensus sequence [46] near the C-terminus of the *STE18* gene (HTY072) [45]. All strains except for RJD360 were derived from RJD863, which originated from W303a. See Table 2 for strain genotypes.

Here we note that our isolate of the RJD863 strain contained a A to G sequence polymorphism at position 2630 of *STE5* compared to the genome sequence in SGD (*Saccharomyces cerevisiae* Genome Database). This polymorphism resulted in a D877G amino acid substitution in the Ste5p protein. However, we did not detect any differences in sensitivity to *α*-factor (Halo Assay), transcriptional activity (*P_FUS1_-GFP* expression), or morphology between strains containing the wild-type Ste5p and strains containing the D877G variant.

We constructed the alternative inputs expression plasmids as follows. Genes in the *α*-factor transcription pathway (*STE2*, *STE4*, *STE5*, *STE5-CTM*, *STE11*, *STE11ΔN* (residues 344–717) *STE7*, *FUS3*, *KSS1*, and *STE12*) were amplified by PCR (Phusion polymerase, New England Biolabs), and then were inserted into the pYES2 or pYES3/CT vectors (Invitrogen) to create the *GAL1* promoter-regulated constructs in a high-copy number plasmid. The *P_GAL1_-STE2^P258L S259L^* and *P_GAL1_-FUS3^I161L^* constructs were created using QuickChange II Site-Directed Mutagenesis Kit (Stratagene). See Table 3 for plasmid constructs.

### Induction of alternative inputs

Cells were grown in selective synthetic media containing 2% dextrose overnight. 0.25 OD_600_ units of cells were harvested, resuspended into 2 ml of selective synthetic media containing 2% raffinose supplemented with adenine, grown for 3 hours, and then 2% galactose (or 2% galactose+1 µM α-factor) was added for 4 hours (for short-term experiments) or 24 hours (for long-term experiments).

### Mating transcriptional activity assay

1.5 ml of the total 2 ml cell culture was harvested and resuspended in PBS. Then, 100 µl of cells was placed into a 96-well plate and transcriptional activation was measured without fixation. The OD_600_ of the cells in the PBS solution was also measured using a spectrophotometer. Mating transcriptional activity from a integrated genomic reporter gene (*P_FUS1_-GFP*) was assayed using a Gemini XS SpectraMAX fluorometer with the excitation at 470 nm and emission at 510 nm as described previously [45]. The GFP fluorescence (arbitrary units) was normalized to the OD_600_, and the *P_FUS1_-GFP*/OD_600_ values were averaged over at least three independent experiments.

### Microscopy

0.4 ml of the total 2 ml cell culture was fixed with ice-cold formaldehyde-PBS solution (3.7% formaldehyde in PBS) for 1 hour. For F-actin staining, cells were fixed with ice-cold formaldehyde-PBS solution for 30 minutes, washed, harvested, and resuspended in PBS with rhodamine-conjugated phalloidin for another 30 minutes, harvested, washed, and resuspended in PBS. Then, 1.5 µl of cells were mounted on a slide with 1 µl of Vectashield mounting solution.

The prepared slides were observed using a Nikon ECLIPSE TE300 fluorescence microscope, and the images were taken by a Hamamatsu ORCA-II CCD camera controlled by the MetaMorph software package.

### Image analysis

In control cells (HT064 (WT), +Gal, t = 24 h), there were no cells possessing a diameter greater than 10 µm; the average diameter was approximately 5 µm. We defined a cell with a diameter greater than 10 µm to be a large cell, and we defined a responding cell to be a large cell or a polarized cell (either elongated or possessing projections); the polarized phenotypes could be determined readily by eye. Most alternative inputs (AI-Ste4p, AI-Ste5p, AI-Ste11p, AI-Ste7p and AI-Ste12p) induced dramatic changes in morphology, so these criteria worked well to distinguish between responding and non-responding cells. For AI-Ste2p and AI-Fus3p cells, we concluded that their phenotypes were small round cells (non-responding).

For counting the number of projections (Figure 6C), we counted at least 100 responding cells. For the morphology AIs-Deletions matrix (Figure 5), we counted responding cells. In these experiments, if fewer than 1% of the total cells were responding cells, then we concluded that they were small round (non-responding) cells. The differences between responding cells and non-responding cells were dramatic. In the future, the morphological classifications would be expedited by automated image analysis.

## Supporting Information

Text S1 Multiple projections induced by AI-Ste12p
(0.02 MB DOC)

Figure S1 Overexpression of wild-type signaling molecules in the α-factor transcription pathway. (A) Transcriptional activation induced by wild-type signaling molecules. Either α-factor (1 µM) was added or the wild-type signaling molecules were induced and transcriptional activation was measured at t = 24 h. Overexpression of Ste2p, Ste11p, Ste7p, Fus3p, and Kss1p did not activate transcription above basal levels. *P_FUS1_-GFP*/OD_600_ values were averaged from at least three measurements, and bar graphs show mean±SEM. (B) The morphologies produced by overexpressing wild-type signaling molecules. Bright field images taken at t = 24 h for a typical set of cells for each wild-type signaling molecule. The scale bar represents 10 µm.
(1.96 MB EPS)

Figure S2 AI-Ste12p in the wild-type (*MFa2+*) strain background produced α-factor. (A) Transcriptional activation induced by AI-Ste12p from both the original strain background (*mf α 1 Δ MFa2+*) and an *mf α 2 Δ* background (*mf α 1 Δ mf α 2 Δ*). *P_FUS1_-GFP*/OD_600_ values were averaged from at least three measurements, and bar graphs show mean±SEM. (B) Bright field images taken at t = 24 h of AI-Ste12p cells in both the original strain background (*mf α 1 Δ MFa2+* and an *mf α 2 Δ* background (*mf α 1 Δ mf α 2 Δ*). The scale bar represents 10 µm. (C) To test whether AI-Ste12p in the (*MFa2+* background (“wild-type”) produced α-factor, we mixed cells (HTY091) containing selected AIs (and no transcriptional reporter) with a *MAT* ***a*** *bar1Δ* reporter strain containing the *P_FUS1_-GFP* construct (HTY146). GFP fluorescence of the reporter strain provided a measure of the α-factor produced by the AI strain. Control cells contained the pYES2 vector, and the result was a basal level of *P_FUS1_-GFP*. The same was true for the AI-Ste4p and AI-Ste7p cells. On the other hand, AI-Ste12p induced significant levels of GFP through the production of α-factor. *P_FUS1_-GFP*/OD_600_ values (t = 24 h) were averaged from at least three measurements, and bar graphs show mean±SEM.
(1.48 MB EPS)
