# Supplementary material for: Synthetic Morphology Using Alternative Inputs
Source: PLoS One. 2009 Sep 10;4(9):e6946. doi: 10.1371/journal.pone.0006946 (PMC2735001; doi:10.1371/journal.pone.0006946)
Supplement: Text S1 — Multiple projections induced by AI-Ste12p (0.02 MB DOC) [file pone.0006946.s001.doc]

# Supplemental Information

# Multiple projections induced by AI-Ste12p

AI-Ste12p induced multiple projections almost identical to wild-type cells treated with α-factor, however, we were able to demonstrate that these multiple projections were caused by α-factor itself which was strongly induced by AI-Ste12p through the *MFα2* gene (Figure S2). The strain background in these experiments were *mfα1Δ MFα2^+^*, and we were able to show that AI-Ste12p cells did indeed make α-factor whereas AI-Ste4p and AI-Ste7p, both of which produced a substantial transcriptional response, did not. Most importantly, when we deleted *MFα2*, the AI-Ste12p cells no longer made multiple projections, but instead were large and round (Figure S2B). The morphology induced by AI-Ste12p through the *Mfα2* gene was surprising and interesting, however, we did not pursue this phenotype because we were interested in producing multiple projections without α-factor.

Control experiments with the other AIs showed no differences caused by the absence of *MFα2* (data not shown).
